# Light Therapy for Myopia Prevention and Control: A Systematic Review on Effectiveness, Safety, and Implementation

**DOI:** 10.1167/tvst.13.8.31

**Published:** 2024-08-21

**Authors:** Dylan James Chang, Sriram P. L., Jooyeon Jeong, Seang-Mei Saw, Nick Sevdalis, Raymond P. Najjar

**Affiliations:** 1 Yong Loo Lin School of Medicine, National University of Singapore, Singapore; 2 Singapore Eye Research Institute, Singapore; 3 Ophthalmology and Visual Science Academic Clinical Program, Duke-NUS Medical School, Singapore; 4 Saw Swee Hock School of Public Health, National University of Singapore, Singapore; 5 Centre for Behavioural and Implementation Science Interventions (BISI), Yong Loo Lin School of Medicine, National University of Singapore, Singapore; 6 Centre for Holistic Initiatives for Learning and Development, Yong Loo Lin School of Medicine, National University of Singapore, Singapore; 7 Eye N' Brain Research Group, Department of Ophthalmology, Yong Loo Lin School of Medicine, National University of Singapore, Singapore; 8 Department of Biomedical Engineering, College of Design and Engineering, National University of Singapore, Singapore; 9 Centre for Innovation & Precision Eye Health, Yong Loo Lin School of Medicine, National University of Singapore, Singapore

**Keywords:** myopia, light therapy, implementation, children, safety

## Abstract

**Purpose:**

This systematic review focuses on the effectiveness, safety, and implementation outcomes of light therapy as an intervention to prevent or control myopia in children.

**Methods:**

A systematic literature search was performed in PubMed, EMBASE, CINAHL, SCOPUS, and Web of Science up to January 27, 2024. Effectiveness outcomes included myopia incidence, and changes in axial length (AL), spherical equivalent refraction (SER), and choroidal thickness (CT). Safety outcomes relating to retinal health or damage and implementation outcomes including compliance rates and loss to follow-up were extracted. ROBINS-I, ROB 2, and ROB-2 CRT were used to assess risk of bias.

**Results:**

Nineteen interventional studies were included. Increased outdoor time (*n* = 3), red-light therapy (*n* = 13), and increased classroom lighting (*n* = 1) had a significant effect on myopia incidence, and changes in AL, SER, and CT. Violet-light therapy (*n* = 2) was only effective in children aged 8 to 10 years and children without eyeglasses with less than 180 minutes of near-work time daily. Two studies using red-light therapy reported adverse effects. For all studies, only compliance rates and loss to follow-up were reported on implementation effectiveness.

**Conclusions:**

Evidence is compelling for the effectiveness of red-light therapy and outdoors time; more data are needed to confirm safety. Robust data are still needed to prove the effectiveness of violet-light and increased classroom lighting. Clearer implementation strategies are needed for all light therapies.

**Translational Relevance:**

Light therapy has emerged as effective for myopia prevention and control. This systematic review summarizes the state of knowledge and highlights gaps in safety and implementation for these strategies.

## Introduction

Myopia, or near-sightedness, affects approximately 2 billion people globally.^1^ It often begins in childhood, progressing throughout adolescence, and is predominantly caused by a mismatch between the optical powers of the cornea and the crystalline lens, and the axial length (AL) of the eye whereby light entering the eye is focused anterior to the retina, leading to the inability to see distant objects clearly.^2^ Although genetics play a role in myopia development,^3^ the rapid surge in myopia prevalence worldwide, and especially in Asia, occurring without significant generational genetic changes, suggests a considerable role of environmental and lifestyle factors in myopia development.^4^

Compared with individuals with mild or moderate myopia, those with high myopia (refractive error of ≤−5 diopters [D]),^5^ are at significantly increased risk for developing complications that may lead to permanent loss of vision.^6^ Some complications include glaucoma, retinal detachment, and cataracts.^6^^,^^7^ Current studies suggest that 9.8% of the world population will have high myopia by 2050.^8^ Several strategies currently aim to manage myopia incidence and progression in children, including atropine eye drops^9^ and orthokeratology.^10^ Single vision optical interventions, like glasses and contact lenses, offer correction but do not necessarily slow progression.^11^

Numerous cross-sectional and longitudinal studies have reported a significant association between increased time spent outdoors and reduced myopia prevalence.^12^^–^^15^ Some of the features of the outdoors that may be protective against myopia are the level and spectral compositions of daylight (i.e. high light levels, broad spectral distribution, and containing infrared [IR] and ultraviolet [UV] light wavelengths),^16^^–^^18^ which are currently lacking in most indoor environments, especially in schools. This, in a way, drove the development of behavioral interventions to increase outdoor time but also the development of light therapy strategies that utilize some of the features of the light outdoors to treat or prevent myopia.

Although increasing the time outdoors remains the most affordable approach to get exposed to daylight,^19^^–^^21^ light therapy interventions in the form of devices (red-light therapy^22^^–^^34^), violet/UV light therapy,^35^^,^^36^ or architectural interventions (e.g. increased classroom lighting^37^) are emerging as potential complementary strategies for myopia prevention and control in children.

Overall, the field of light therapy remains a novel and emerging field of study. The majority of light interventions, particularly those using violet light, lack comprehensive reviews on their effectiveness in reducing myopia progression. While red-light therapy is relatively more established with various systematic reviews and meta-analyses^38^^–^^41^ on its effectiveness in slowing myopia progression, it has yet to be tested for myopia prevention. Furthermore, there is a lack of systematic understanding of implementation strategies for red-light therapy, in contrast with successful implementation strategies for interventions involving increased outdoor time.^42^^–^^44^

This systematic review addresses these gaps in the evidence base. Our primary aim was to provide a comprehensive overview of effectiveness and safety of light therapies for myopia prevention and control, including red light, violet light, outdoor time, and improved classroom lighting. Our secondary aim was to synthesize evidence on implementation strategies for such interventions.

## Methods

The review was conducted following the Preferred Reporting Items for Systematic Reviews and Meta-Analyses (PRISMA) checklist^45^ and guidance from the Cochrane Handbook of Systematic Reviews of Interventions.^46^ The protocol of the review was registered on the International Prospective Register of Systematic Reviews (PROSPERO) under ID CRD42024513404 on February 16, 2024.

### Literature Search

A literature search on Embase, Medline (PubMed), Scopus, Web of Science, and CINAHL (latest search performed on January 27, 2024). The full search strategy can be found in the online supplementary material (Supplementary Table S1). Both medical subject heading (MeSH) terms and general keywords related to “myopia” and “light” were used as search terms. This search strategy was developed through discussions between the authors and consultations with an expert librarian from the National University of Singapore, Singapore.

Titles and abstracts of the papers found were independently reviewed by pairs of reviewers (authors D.C., P.S., and J.J.; Pairs: D.C. and P.S., D.C. and J.J., and P.S. and J.J.) and the relevant papers were identified. A full text analysis was carried out on all the relevant papers to identify those that were suitable to be included in this review. Any disagreements between the reviewers on the papers included were resolved by group discussions with authors Nick Sevdalis and Raymond P. Najjar and reviews of the papers within the author group, including the two senior authors (R.P.N. a visual neurosciences research expert and N.S. an implementation science expert). Additionally, the reference lists for each shortlisted paper were also analyzed to identify papers of relevance to this review.

### Eligibility Criteria

All types of interventional studies, including randomized controlled trials, non-randomized controlled trials, and case reports, were included to allow for a comprehensive analysis of the efficacy, safety, and implementation aspects of light therapies. We included studies that used artificial and natural light exposure as an intervention to prevent myopia and/or control its progression.

Specifically, studies that investigated the effectiveness of any form of intervention involving the direct use of light on myopia prevention and/or control in children and adolescents aged 5 to 18 years were included. Studies that conducted similar analyses on the same study population were compared and the study most relevant to the research question was selected for inclusion in the systematic review. Secondary studies, such as reviews of any type or meta-analyses and observational studies were excluded. Articles not reporting primary data, such as letters to the editors or commentaries, papers in languages other than English, and papers not reporting data directly related to our research questions or were available in abstract-only format were also excluded. Studies that did not involve human subjects, did not measure or report myopia progression in quantitative terms (e.g. using measures of SER or AL), and did not mention prevention or control of myopia were excluded. Additionally, studies that focused on treatment of myopia symptoms (e.g. refractive surgeries) and observational studies that did not detail an intervention to prevent myopia progression were excluded. There was no date restriction applied to the studies we considered.

### Data Extraction and Quality Assessment

Data extraction was carried out by pairs of reviewers (authors D.C. and P.S. and J.J.; Pairs: D.C. and P.S., D.C. and J.J., and P.S. and J.J.) and a data extraction table was crafted in an Excel 2016 (Microsoft, USA) spreadsheet to extract the relevant data from the articles. Any disagreements between the reviewers were resolved by group discussions and reviews of the papers, including the senior review authors (R.P.N. and N.S.).

Quality assessment was performed using the Risk Of Bias In Non-Randomized Studies - of Interventions (ROBINS-I) tool, the Cochrane Risk Of Bias (ROB 2) tool, and revised Cochrane risk of bias tool for cluster randomized trials (ROB-2 CRT). Risk assessment was performed independently by pairs of reviewers (authors D.C. and P.S. and J.J.) with any disagreements being resolved through discussion. ROBINS-I is an assessment tool designed for evaluating the risk of bias in estimates of the comparative effectiveness (harm or benefit) of interventions from studies that did not use randomization to allocate units (individuals or clusters of individuals) to comparison groups.^47^ ROB 2 is a tool designed specifically for identifying the risk of bias in randomized trials,^48^ whereas ROB-2 CRT is a tool used for cluster randomized trials.^48^ The characteristics assessed by the tools used were methods of randomization, timing of recruitment of participants, deviations from intended intervention, missing data, measurement of outcomes, selection of reported results, and confounding and classification of interventions. The ROB-2 and ROB-2 CRT tools judged studies overall to be of low risk, having some concerns or high risk of bias; the ROBINS-I tool judged studies overall to be of low risk, moderate risk, serious risk, critical risk of bias, or having no information to base a judgment of risk of bias. Studies that were assigned a low risk of bias were considered to have taken appropriate measures to minimize bias and its results were taken to be reliable. Studies with moderate risk of bias were deemed to have methodological flaws and limitations that could reduce the reliability of the results; however, these limitations were not severe enough to invalidate the studies’ findings. A study with a high risk of bias was one with significant limitations in its study design, methodology, or analysis which could undermine the validity of its results.^48^ Additionally, a study with serious risk of bias suggested that the study had significant concerns that may affect the validity of its results and a study with a critical risk of bias was one with the most severe flaws which make its results unreliable to make informed decisions.^47^ Tabulation and visual presentation of risk of bias assessment results for the ROB 2, ROB-2 CRT, and ROBINS-I tools were made using the ROBvis tool.^49^

### Data Synthesis and Analysis

A qualitative data synthesis was performed to summarize and analyze the following study characteristics: details of intervention, primary effectiveness outcomes (i.e. myopia incidence, and changes in SER and AL), secondary effectiveness outcomes (i.e. changes in CT), safety outcomes (e.g. reports of glare, flash blindness, afterimages longer than 6 minutes after treatment, functional damage as indicated by best corrected visual acuity [BCVA], and no abnormalities in the retinal or choroidal structure), and implementation outcomes associated with the intervention (e.g. compliance rate) extracted as per the established taxonomy.^50^ In addition, we also extracted characteristics of the intervention relating to its implementability – specifically factors negatively affecting implementation and strategies to support or optimize implementation.

## Results

### Search Results

A total of 8175 papers were identified through the following search strategy, which can be accessed in full in the online supplementary material (see Supplementary Table S1). Following removal of duplicate papers and untitled papers with no attached abstracts using Endnote 21 (Clarivate, USA), 5356 papers remained. After screening by title and abstract, 42 papers were highlighted for full text review. After screening the full text of the 42 papers, 19 observational studies^44^^,^^51^^–^^68^ and one retrospective case series^69^ were excluded. In addition, two papers conducted similar analyses on the same study populations and offered no novel insight,^70^^,^^71^ one paper focused on a population which did not meet the inclusion criteria,^72^ one paper described an intervention without a control group,^73^ and four papers did not measure or report myopia progression in quantitative terms.^74^^–^^77^ A further six papers were found through searching the citations of studies which met the inclusion criteria. Out of these six papers, only one paper was excluded that had a full text which was not in English.^78^ At the final screening stage, 19 interventional studies^19^^–^^37^ were included in the systematic review. The final PRISMA flow diagram for the review is shown in the Figure.

**Figure.**
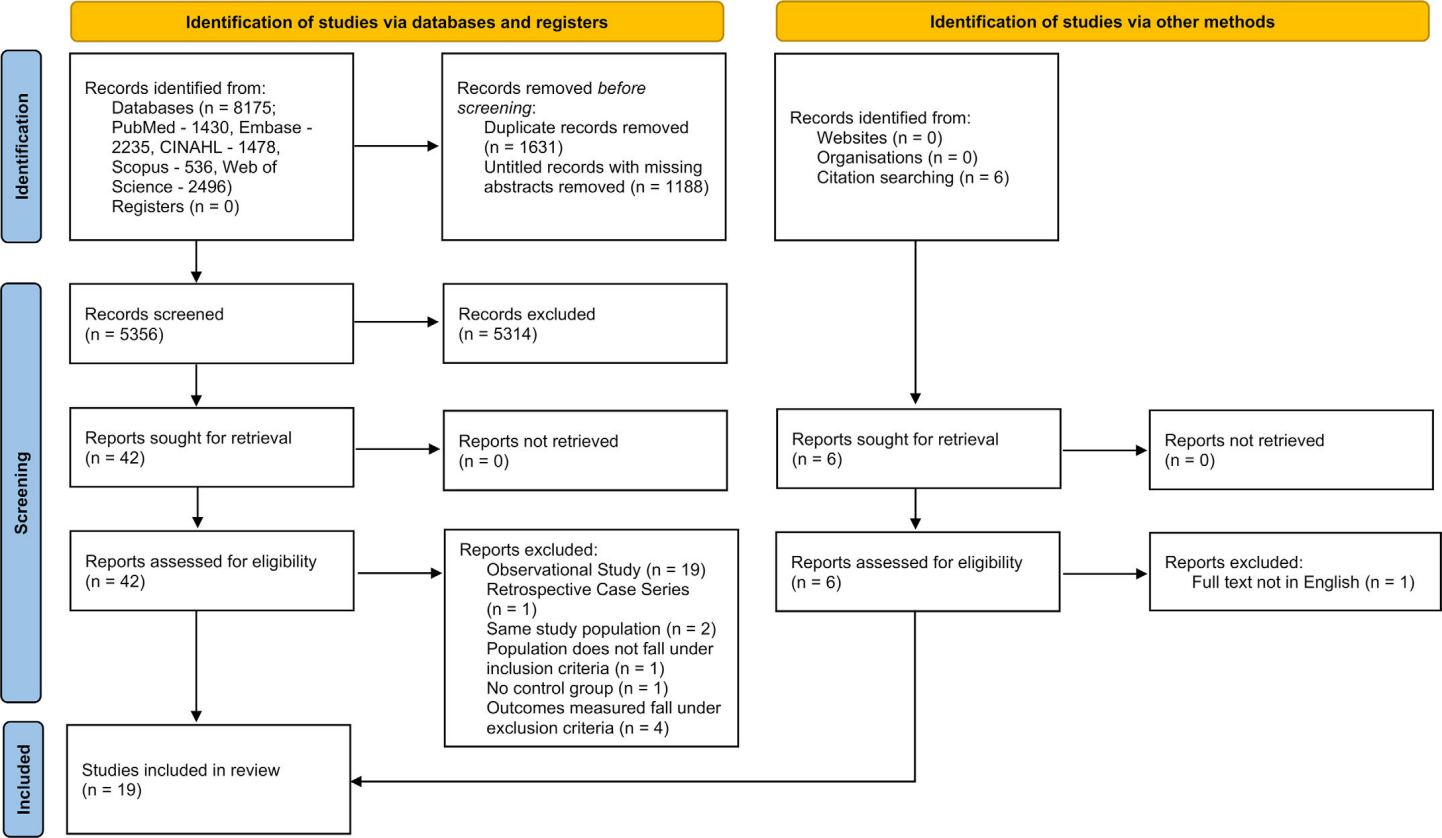
PRISMA flow diagram depicting the screening process for the review.

### Description of Included Studies

The characteristics of the 19 selected studies and details of their interventions are summarized in Table 1. Our review included randomized controlled trials, non-randomized interventional studies, and a post-trial follow up real world study of a previous randomized controlled trial. Studies focused on red-light therapy (*N* = 13), violet light therapy (*N* = 2), increased outdoor time (*N* = 3), and improved indoor classroom lighting (*N* = 1) as interventions for myopia prevention and control. The majority of studies measured myopia prevention and control quantitatively through measures of AL (*N* = 16) and SER (*N* = 17). A smaller number of studies also measured changes in CT (*N* = 11) and differences in myopia incidence (*N* = 5). Of the 19 selected studies, 17 studies measured cycloplegic SER, whereas 2 studies using red-light therapy measured non-cycloplegic SER.

**Table 1.** Characteristics of Included Studies

Author, Year; Study Design; Sample Size	% of Males, Ethnicity	Age Range, Years, Refraction	Details of Intervention	Duration of Last Follow-Up	Clinical Effectiveness Outcomes Measured
He et al., 2023^23^; RCT; *N* = 278	50%, Chinese	6 y – 11 y	Low-level 650 nm red-light therapy twice daily, 5 d a week, 3 min per session, intervals of 4 h between sessions	12 mo	Myopia incidence; changes in AL, cycloplegic SER, CT, UCVA, BCVA, and vision function
Chen et al., 2023^22^; RCT; *N* = 102	58%, Chinese	6 y – 13 y	Low intensity 635 nm red light therapy with a power of 0.35 ± 0.02 mW twice daily for 3 min each session, with at least a 4-h interval between sessions	12 mo treatment, 3 mo post-treatment follow-up to assess rebound	Changes in AL, cycloplegic SER, SFCT, BCVA, accommodative function and ocular anatomic structure
Dong et al., 2023^24^; RCT; *N* = 112	50.4%, Chinese	7 y – 12 y	Low-level red-light therapy of either RLRL device (0.29 mW) or sham device (0.03 mW) twice daily, for 3 min per session, with an interval between sessions of at least 4 h	6 mo	Changes in AL, cycloplegic SER and UCVA
Jiang et al., 2022^25^; RCT; *N* = 264	49.2%, Chinese	8 y – 13 y	Repeated low-level red-light therapy of 650-nm wavelength with a power of 0.29 mW at an illuminance level of approximately 1600 lux, administered at home under supervision of parents twice daily, 5 d per week, for 3 min per session, with a minimum interval of 4 h between sessions	12 mo	Changes in AL, cycloplegic SER, CT, and UCVA
Liu et al., 2024^26^; RCT; *N* = 94	54.1%, Chinese	7 y – 12 y	Repeated low-level 650 nm red-light therapy treatment with a power of 0.29 mW at an illuminance level of approximately 1600 lux twice daily, with each session lasting 3 min with a minimum interval of 4 h between sessions	12 mo	Myopia incidence; changes in AL, cycloplegic SER, CT, RT, superficial retinal vascular density, deep retinal vascular density, choriocapillaris perfusion area, and choroidal vessel volume in the foveal, parafoveal, and perifoveal regions
Lin et al., 2023^27^; RCT; *N* = 210	48.8%, Chinese	6 y – 12 y	Repeated low-level 650-nm red-light therapy with laser power of 2 ± 0.5 mW using the instrument at home twice a day, 5 d a week, for 3 min each session with a minimum interval of 4 h between sessions; after each treatment participants closed their eyes and rested for 2 min	60 d	Changes in AL and non-cycloplegic SER
Yang et al., 2022^28^; RCT; *N* = 25	Not reported, Chinese	6 y – 12 y	Low-level 650 ± 10 nm red-light therapy with a power of 0.16 mW of approximately 1600 lux was used for 3 min	1 h after treatment	Changes in CFT, retinal fovea thickness, retinal foveal perfusion, and choroidal fovea blood flow
Wang et al., 2023^29^; non-randomized controlled trial; *N* = 49	65.9%, Chinese	6 y – 12 y	Low-level 650 nm red-light with a power of 0.60 mw at an illuminance range of roughly 1200–1800 lux, with therapy given at home under parental guidance twice daily, 7 d per week, for 3 min each session with a minimal interval of 4 h between sessions	3 mo	Changes in AL, cycloplegic SER and SFCT
Xiong et al., 2022^30^; post-trial follow up real world study (RWS) of Jiang et al., 2022; *N* = 138	46.5%, Chinese	8 y – 13 y	Repeated low-level red-light therapy (650-nm) at a power of 0.29 mW and an illuminance of approximately 1600 lux, administered at home under supervision of parents twice daily, 5 d per week for 3 min per session, with a minimum interval of 4 h between sessions; same as Jiang et al., 2022	24 mo after Jiang et al., 2022 RLRL RCT	Changes in AL, cycloplegic SER, CT, UCVA, BCVA, corneal curvature, and anterior chamber depth
Zhou et al., 2024^31^; RCT; *N* = 200	55%, Chinese	6 y – 15 y	Repeated low-level 650 nm red-light therapy at 0.37 mW, 0.60 mW, and 1.20 mW administered at home under the supervision of their parents twice daily for 3 min per session, with a minimum interval of 4 h between sessions	6 mo	Changes in AL, non-cycloplegic SER and SFCT
Zhao et al., 2023^32^; non-randomized controlled trial; *N* = 67	61.2%, Chinese	6 y – 14 y	Repeated low-level 650 nm red-light therapy at a power of 2 mw administered at home twice daily for 3 min per session, with a minimum interval of 4 h between sessions	4 wk	Changes in SFCT, RT, total choroidal area, luminal area, stromal area, choroidal vascularity index, percentage retinal vascular density, and choriocapillaris flow voids
Xiong et al., 2021^33^; RCT; *N* = 200	54%, Chinese	7 y – 14 y	Low-level 650 nm red-light therapy at a power of 2 ± 0.5 mW twice daily for 3 min each session, with a minimum interval of 4 h between sessions	6 mo	Changes in AL, SER, and SFCT
Tian et al., 2022^34^; RCT; *N* = 224	50%, Chinese	6 y – 12 y	Low-level 650 nm red-light therapy twice daily for 3 min each session, with a minimum interval of 4 h between sessions	6 mo	Changes in AL, cycloplegic SER, CT, flat keratometry, and change in steep keratometry
Mori et al., 2021^35^; RCT; *N* = 113	38.1%, Japanese	6 y – 12 y	Wearing violet light glasses that transmit 360–400 nm light versus wearing conventional eyeglasses that did not transmit violet light	24 mo	Changes in AL and cycloplegic SER
Torii et al., 2022^36^; RCT; *N* = 43	60.5%, Japanese	6 y – 12 y	Wearing of violet light emitting (360 – 400 nm, 310 µW/cm2) eyeglass frames versus pseudo-placebo eyeglasses for the control group with a minimal amount of violet light irradiance of less than 10 µW/cm2 for 3 h daily in both groups from 11 AM to 2 PM	24 wk	Changes in AL and cycloplegic SER
Hua et al., 2015^37^; RCT; *N* = 317	47.9%, Chinese	6 y – 14 y	Lighting systems were rebuilt in every intervention classroom parallel to the window in two rows, and a separate blackboard lamp fixture with one tube was installed over the front blackboardMedian illuminance of desks after intervention – 558 luxMedian illuminance of blackboards after intervention – 440 luxMedian illuminance of desks in control group – 98 luxMedian illuminance of blackboards in control group – 76 lux	1 y	Myopia incidence; changes in AL, cycloplegic SER, corneal curvature, and anterior chamber depth
He et al., 2022^19^; RCT; *N* = 6295	Not reported, Chinese	6 y – 9 y	Control versus additional outdoor time of 40 min per school day versus additional 80-min outdoor time per school day	24 mo	Myopia incidence; changes in AL and cycloplegic SER
Wu et al., 2018^20^; RCT; *N* = 930	52.2%, Chinese	6 y – 7 y	Participants were encouraged to have 11 h or more of outdoor time every 7 d. Children were encouraged to take a 10-min break from every 30 min of near work. Family weekend outdoor activities were encouraged through, routine learning assignments, honor rewards for students, and local upcoming outdoor family event information for outdoor activities and near-work breaks	1 y	Myopia incidence; changes in AL and cycloplegic SER
Wu et al., 2013^21^; non-randomized controlled trial; *N* = 571	51.6%, Chinese	7 y – 11 y	Recess outside the classroom program in which the classroom lights were turned off, classrooms were emptied, and all children were encouraged to go outside of the classroom for 80 min of outdoor activities during recess time	1 y	Myopia incidence; changes in cycloplegic SER

AL, axial length; BCVA, best-corrected visual acuity; CT, choroidal thickness; CFT, choroidal foveal thickness; RCT, randomized controlled trials; RT, retinal thickness; SER, spherical equivalent refraction; SFCT, subfoveal choroidal thickness; UCVA, uncorrected visual acuity.

### Summarized Description of Interventions

In studies using red-light therapy, low-level red-light ranging from 635 to 650 nm was administered twice daily, 5 to 7 days a week, for 3 minutes per session with intervals of 4 hours between 2 sessions using a 0.03 to 2.00 mW light source with an illuminance range of 1200 to 1800 lux.^22^^–^^34^

He et al.’s study in 2022,^19^ involved providing 40 or 80 minutes of additional outdoor time per school day to participants. Wu et al., in 2013,^21^ increased the recess time spent outdoors by emptying classrooms, turning classroom lights off, and by encouraging all children to participate in outdoor activities during recess time. Wu et al.’s study in 2018,^20^ increased outdoor time by encouraging students to take a 10-minute break for every 30 minutes of near work activities, by encouraging students to have 11 hours or more of outdoor time every 7 days, and also by encouraging family weekend outdoor activities through routine learning assignments, honor rewards for students, and local upcoming outdoor family event information to incentivize outdoor activities and near-work breaks.

Mori et al.’s study in 2021,^35^ involved participants wearing violet light glasses that transmitted 360 to 400 nm light for 24 months, compared to a control group wearing conventional eyeglasses that did not transmit violet light, whereas the study by Torii et al., in 2022,^36^ involved participants wearing violet light emitting eyeglass frames at an irradiance of 310 µW/cm^2^ compared to a control group wearing pseudo-placebo eyeglasses for the control group with a minimal amount of violet light irradiance of less than 10 µW/cm^2^, with violet light irradiation occurring for 3 hours daily in both groups from 11 AM to 2 PM*.*

The study by Hua et al. in 2015,^37^ rebuilt lighting systems in classrooms to allow for a minimum average illuminance of 300 lux on desks and 500 lux on blackboards. Eight suspension-mounted grille luminaires with 16 fluorescent tubes of 6500 K were hung from the ceiling parallel to the window in two rows, and a separate blackboard lamp fixture with one tube was installed over the front blackboard. Furthermore, fluorescent lights near the interior wall were lit up on sunny days to avoid relative dimness compared to the areas beside the windows.

### Assessment of Risk of Bias

Ten of the randomized studies included were judged to have a low risk of bias across all domains.^21^^,^^23^^,^^25^^,^^26^^,^^28^^,^^30^^,^^31^^,^^33^^,^^35^^,^^36^ However, the study by Dong et al., in 2023,^24^ raised some concerns due to the selection of the reported result: the study report offered no information regarding data produced being analyzed in accordance with a prespecified analysis plan that was finalized before unblinded outcome data were available for analysis. The studies by Chen et al., in 2023,^22^ and Tian et al., in 2022,^34^ were found to be of high risk of bias due to deviations from intended interventions. This is because, for both studies, participants, carers, and people delivering the interventions were aware of the intervention groups during the trial and no information was presented about deviations from the intended intervention because of the trial context. Furthermore, “naïve” per-protocol analysis was used which was inappropriate to estimate the effect of adhering to the intervention (Supplementary Fig. S1).

Of the cluster randomized controlled trials, the studies by Wu et al., in 2018,^20^ and He et al., in 2022,^19^ had some concerns, whereas Hua et al., in 2015,^37^ had high risk of bias due to deviations from intended interventions. In the studies by Wu et al., in 2018,^20^ and He et al., in 2022,^19^ the concerns were mainly due to the presence of non-adherence to the assigned intervention, which would have affected the participants’ outcomes. In the study by Hua et al., in 2015,^37^ deviations arose from the trial context with the lighting condition of blackboards not improving as much as expected due to physical limitations, combined with the lack of information as to whether this deviation was balanced between test groups (Supplementary Fig. S2).

Three non-randomized interventional studies were judged to have serious risks of bias. The studies by Lin et al., in 2023,^27^ Wang et al., in 2023,^29^ and Zhao et al., in 2023,^32^ had potential biases due to confounding factors such as age, time spent doing near work, parental myopia, compliance to treatment, gender, baseline SER, and baseline AL. The study by Lin et al., in 2023,^27^ also had high risk of bias from missing data due to substantially different proportions of missing participants across groups (Supplementary Fig. S3).

Current evidence supports the effectiveness of red-light therapy for myopia control and outdoor time for myopia prevention. Yet, some adverse events were associated with red-light therapy, and data on the implementation of all light therapies are limited. The following sections detail the effectiveness, safety, and implementation findings for all interventions.

## Interventional Studies Using Red Light Therapy

### Effectiveness

Thirteen studies used red-light therapy as an intervention and showed a significant reduction in axial elongation (*P* < 0.05) between the intervention and control groups after follow-ups ranging from 4 weeks^32^ up to 24 months of treatment with the intervention^30^ (Table 2). Evidence of significant axial shortening compared to baseline measurements was reported after 3 months,^29^ 6 months,^22^^,^^24^^,^^34^ and up to 12 months of red-light therapy.^25^ Ten studies showed a significantly slower SER progression,^22^^–^^26^^,^^29^^–^^31^^,^^33^^,^^34^ whereas only one study showed no significant difference in SER after treatment compared with the control groups^27^ (see Table 2). Nine studies showed a significant increase in subfoveal CT after red-light therapy,^22^^,^^23^^,^^26^^,^^29^^–^^34^ whereas one study showed no significant changes in CT 1 hour after red-light therapy^28^ and one study reported changes in CT with no associated *P* value^25^ (see Table 2).

**Table 2.** Effectiveness and Safety Outcomes

		Reported Data on Clinical Effectiveness Outcomes (Intervention Group Vs Control Group)				
Type of Intervention	Author, Year; Study Design; Sample Size	Change in Axial Length, mm	Change in Spherical Equivalent Refraction, D	Change in Choroidal Thickness, µm	Myopia Incidence	Reported Data of Clinical Safety Outcomes
Red light	He et al., 2023^23^; RCT; *N* = 278	Intervention = +0.30 ± 0.27Control = +0.47 ± 0.25Absolute mean difference = +0.17(95% CI = 0.11–0.23)*P* < 0.001	Intervention = −0.35 ± 0.54Control = −0.76 ± 0.60Absolute mean difference = −0.41 (95% CI = −0.56 to –0.26)*P* < 0.001	Intervention = +3.0 ± 16.9Control = −9.2 ± 22.3*P* < 0.001	Intervention = 40.8%Control = 61.3%Absolute mean difference = 20.4% (95% CI = 7.9%–33.1%)*P* = 0.003Relative risk = 0.67 (95% CI = 0.51–0.86)	No functional damage or adverse effects reported
Red light	Chen et al., 2023^22^; RCT; *N* = 102	*First 12 mo*Intervention = +0.01 (95% CI = −0.05 to 0.07)Control = +0.39 (95% CI = 0.33 to 0.45)*P* < 0.05*3 mo rebound*Intervention (compared with dataat the time of treatmentcessation) = +0.16 (95% CI = 0.11 to 0.22)*P* < 0.05	*First 12 mo*Intervention = +0.05 (95% CI = −0.08 to 0.19)Control = −0.64(95% CI = −0.78 to −0.51)*P* < 0.05*3 mo rebound*Intervention (compared with dataat the time of treatmentcessation) = −0.20(95% CI = −0.26 to −0.14)*P* < 0.05	Intervention = +21.57 (95% CI = 12.00 to 31.13)Control = −11.30 (95% CI = −17.37 to −5.23)*P* < 0.05	Not available	No functional damage or adverse effects reported
Red light	Dong et al., 2023^24^; RCT; *N* = 112	Intervention = +0.02 ± 0.11Control = +0.13 ± 0.10*P* < 0.001	Intervention = +0.06 ± 0.30Control = −0.11 ± 0.33*P* = 0.003	Not available	Not available	No functional damage or adverse effects reported relating to RLRL therapy
Red light	Jiang et al., 2022^25^; RCT; *N* = 264	Intervention = +0.13 (95% C = 0.09 to 0.17)Control = +0.38 (95% CI = 0.34 to 0.42)Mean difference = +0.26 (95% CI = 0.20 to 0.31)*P* < 0.001	Intervention = −0.20(95% CI = −0.29 to −0.11)Control = −0.79(95% CI = −0.88 to −0.69)Difference = −0.59(95% CI = −0.72 to −0.46)*P* < 0.001	Intervention = +12.1 (95% CI = 6.1 to 18.1)Control = −9.5 (95% CI = −15.6 to −3.5)No *P* value given	Not available	No functional damage or adverse effects reported
Red light	Liu et al., 2024^26^; RCT; *N* = 94	Intervention = +0.09 ± 0.21Control = +0.28 ± 0.12Difference = 0.19*P* < 0.001	Intervention = −0.11 ± 0.44Control = −0.42 ± 0.36Difference = −0.31*P* < 0.001	Intervention = +11.99 ± 32.66Control = −28.74 ± 26.89*P* < 0.001	Intervention = 20.9%Control = 38.1%Absolute mean difference = 17.2%No *P* value given	Two children reported intolerance to bright light and dry eye symptoms which were transient and resolved either spontaneously or with minimal intervention
Red light	Lin et al., 2023^27^; RCT; *N* = 210	Intervention *(mild to moderatemyopia)* = −0.03 ± 0.11Intervention *(severemyopia)* = −0.07 ± 0.11Control = −0.08 ± 0.40*P* < 0.001	Intervention *(mild to moderatemyopia)* = +0.06 ± 0.37Intervention *(severemyopia)* = +0.06 ± 0.30Control = −0.26 ± 1.91*P* = 0.456	Not available	Not available	Main adverse reaction to the red light delivery instrument was an afterimage which was alleviated by a short period of eye-closing and rest
Red light	Yang et al., 2022^28^; RCT; *N* = 25	Not available	Not available	Intervention = −3.44 ± 2.08Control = −2.08 ± 2.08*P* = 0.635	Not available	Did not assess adverse effects related to red light therapy
Red light	Wang et al., 2023^29^; non-randomized controlled trial; *N* = 49	Intervention = −0.08(95% CI = −0.11 to −0.06)Control = +0.08 (95% CI = 0.05 to 0.11)Difference = +0.17 (95% CI = 0.13 to 0.20)*P* < 0.001	Interventional = +0.23(95% CI = 0.13 to 0.33)Control = −0.07(95% CI = −0.16 to −0.03)Difference = +0.30(95% CI = −0.42 to −0.18)*P* < 0.001	Intervention = +24.21 (95% CI = 14.86 to 33.56)Control = −4.28(95% CI = −15.91 to −7.35)Difference = −27.84 (95% CI = −40.02 to −15.67)*P* < 0.001	Not available	No functional damage or adverse effects reported
Red light	Xiong et al., 2022^30^; post-trial follow up RWS of Jiang et al., 2022; *N* = 138	*Baseline to 24 mo*SVS-SVS = +0.64 ± 0.24SVS-RLRL = +0.44 ± 0.37RLRL-SVS = +0.50 ± 0.28RLRL-RLRL = +0.16 ± 0.37*P values between groups*SVS-SVS versus SVS-RLRL: *P* = 0.366SVS-SVS versus RLRL-SVS: *P* = 0.156SVS-SVS versus RLRL-RLRL: *P* < 0.001SVS-RLRL versus RLRL-SVS: *P* = 1.000SVS-RLRL versus RLRL-RLRL: *P* = 0.206RLRL-SVS versus RLRL-RLRL: *P* = 0.005Overall: *P* < 0.001	*Baseline to 24 mo*SVS-SVS = −1.24 ± 0.63SVS-RLRL = −0.96 ± 0.70RLRL-SVS = −1.07 ± 0.69RLRL-RLRL = −0.31 ± 0.79*P values between groups*SVS-SVS versus SVS-RLRL: *P* = 1.000SVS-SVS versus RLRL-SVS: *P* = 1.000SVS-SVS versus RLRL-RLRL: *P* = 0.001SVS-RLRL versus RLRL-SVS: *P* = 1.000SVS-RLRL versus RLRL-RLRL: *P* = 0.325RLRL-SVS versus RLRL-RLRL: *P* = 0.010Overall: *P* = 0.003	*Baseline to 24 mo*SVS-SVS = −16.09 ± 19.37SVS-RLRL = −2.93 ± 25.64RLRL-SVS = −8.66 ± 24.68RLRL-RLRL = +21.49 ± 36.21*P values between groups*SVS-SVS versus SVS-RLRL: *P* = 1.000SVS-SVS versus RLRL-SVS: *P* = 1.000SVS-SVS versus RLRL-RLRL: *P* = 0.002SVS-RLRL versus RLRL-SVS: *P* = 1.000SVS-RLRL versus RLRL-RLRL: *P* = 0.292RLRL-SVS versus RLRL-RLRL: *P* = 0.012Overall: *P* = 0.004	Not available	No functional damage or adverse effects reported
Red light	Zhou et al., 2024^31^; RCT; *N* = 200	0.37-mW group = +0.04 (95% CI = −0.01 to 0.08)0.60-mW group = 0.00(95% CI = −0.05 to 0.05)1.20-mW group −0.04(95% CI = −0.08 to 0.01)Control = +0.27(95% CI = 0.22 to 0.33)Adjusted *P* < 0.001 for all;adjusted *P* > 0.05 between all comparisonsof different powers	0.37-mW group = +0.01(95% CI = −0.12 to 0.15)0.60-mW group = −0.05(95% CI = −0.18 to 0.07)1.20-mW group = +0.16(95% CI = 0.03 to 0.30)Control group = −0.22(95% CI = −0.50 to 0.30)Adjusted *P* < 0.001 for all;adjusted *P* > 0.05 between all comparisonsof different powers	0.37-mW group = +22.63 (95% CI = 12.13 to 33.34)0.60-mW group = +36.17 (95% CI = 24.37 to 48.25)1.20-mW group = +42.59 (95% CI = 23.43 to 66.24)Control group = −5.07 (95% CI = −10.32 to −0.13)Adjusted *P* < 0.001 for all;adjusted *P* > 0.05 between all comparisonsof different powers	Not available	No functional damage or adverse effects reported
Red light	Zhao et al., 2023^32^; non-randomized controlled trial; *N* = 67	Not available	Not available	Intervention = +14.5 (95% CI = 9.6 to 19.5)Control = −1.7 (95% CI = −9.1 to 5.7)Mean difference = −16.2 (95% CI = 7.8 to 24.7)*P* < 0.0001	Not available	Did not assess adverse effects related to red light therapy
Red light	Xiong et al., 2021^33^; RCT; *N* = 200	Intervention = +25.00 ± 1.11Control = +25.30 ± 0.86*P* < 0.001	Intervention = −3.17 ± 2.14Control = −3.82 ± 1.37*P* < 0.001	Intervention = +323.91 ± 65.63Control = +269.97 ± 64.11*P* < 0.001	Not available	Did not assess adverse effects related to red light therapy
Red light	Tian et al., 2022^34^; RCT; *N* = 224	Intervention = 0.06(IQR = −0.15 to 0)Control = 0.14(IQR = 0.07 to 0.22)*P* < 0.001	Intervention = 0.125(IQR = 0 to 0.375)Control = 0.25(IQR = −0.5 to 0)*P* < 0.001	Intervention = +15(IQR = −3 to 34.5)Control = −7(IQR = −28 to 14.5)*P* < 0.001	Not available	No functional damage or adverse effects reported
Violet light	Mori et al., 2021^35^; RCT; *N* = 113	*No limited conditions* Intervention = +0.728(95% CI = 0.682−0.775)Control = +0.758(95% CI = 0.711−0.810)Mean difference = −0.03 (95% CI = −0.10 to 0.04)*P* = 0.381*Limited analysis to “children who firststarted using eyeglasses” and “nearwork time of less than 180 min”*Intervention = +0.75(95% CI = 0.65 to 0.86)Control = +0.96 (95% CI = 0.86 to 1.06)Mean difference = −0.21 (95% CI = −0.35 to −0.06)*P* = 0.006	*No limited conditions*Intervention = −1.421(95% CI = −1.617 to −1.225)Control = −1.531(95% CI = −1.729 to −1.330)Mean difference = +0.11(95% CI = −0.17 to 0.39)*P* = 0.434*Limited analysis to “children who firststarted using eyeglasses” and “nearwork time of less than 180 min”*Intervention = −1.54 (95% CI = −1.76 to −1.32)Control = −1.84 (95% CI = −2.06 to −1.63)Mean difference = +0.30 (95% CI = −0.01 to 0.61)*P* = 0.055	Not available	Not available	No functional damage or adverse effects reported
Violet light	Torii et al., 2022^36^; RCT; *N* = 43	*Mean change after subgroup analysisof children aged 8−10 y*Intervention = 0.14 ± 0.03Control = 0.23 ± 0.02*P* = 0.016	*Mean change after subgroup analysisof children aged 8–10 y*Intervention = −0.12 ± 0.12Control = −0.60 ± 0.10*P* = 0.008	Not available	Not available	No functional damage or adverse effects reported
Improved classroom lighting	Hua et al., 2015^37^; RCT; *N* = 317	*Non-myopic children*Intervention = +0.13 ± 0.17Control = +0.18 ± 0.12*P* < 0.05*Myopic children*Intervention = +0.20 ± 0.11Control = +0.27 ± 0.10*P* < 0.01	*Non-myopic children*Intervention = −0.25 ± 0.40Control = −0.47 ± 0.40*P* < 0.01*Myopic children*Intervention = −0.25 ± 0.47Control = −0.31 ± 0.46*P* > 0.05	Not available	Intervention = 4%Control = 10%*P* = 0.029	Did not assess adverse effects related to red light therapy
Outdoor light exposure	He et al., 2022^19^; RCT; *N* = 6295	Additional 40 min of outdoor time = +0.55 (95% CI = 0.51–0.60)Additional 80 min of outdoor time = +0.57 (95% CI = 0.52−0.62)Control = +0.65(95% CI = 0.60−0.70)*P* < 0.044 among the three groups	Additional 40 min of outdoor time = −0.84(95% CI = −0.96 to −0.70)Additional 80 min of outdoor time = −0.91(95% CI = −1.03 to −0.79)Control = −1.04(95% CI = −0.91 to −1.17)*P* > 0.05 among the three groups	Not available	Additional 40 min of outdoor time Incidence risk ratio = 0.84(95% CI = 0.72 to 0.99)*P* = 0.035Additional 80 min of outdoor time Incidence risk ratio = 0.89(95% CI = 0.79 to 0.99)*P* = 0.041	Did not assess adverse effects related to red light therapy
Outdoor light exposure	Wu et al., 2018^20^; RCT; *N* = 930	*Non-myopic children*Intervention = +0.26 ± 0.18Control = +0.30 ± 0.32Estimated difference = −0.03 (95% CI = −0.06 to −0.01)*P* = 0.02*Myopic children*Intervention = +0.45 ± 0.28Control = +0.60 ± 0.19Estimated difference = −0.15 (95% CI = −0.28 to −0.02)*P* = 0.02	*Non-myopic children*Intervention = −0.32 ± 0.58Control = −0.43 ± 0.75Estimated difference = +0.11 (95% CI = 0.02−0.20)*P* = 0.02*Myopic children*Intervention = −0.57 ± 0.40Control = −0.79 ± 0.38Estimated difference = +0.23 (95% CI = 0.06−0.39)*P* = 0.007	Not available	Odds ratio = 0.65 (95% CI = 0.42−1.01)*P* = 0.05	Did not assess adverse effects related to red light therapy
Outdoor light exposure	Wu et al., 2013^21^; non-randomized controlled trial; *N* = 571	Not available	*Non-myopic children*Intervention = −0.26 ± 0.61Control = −0.44 ± 0.64*P* = 0.020*Myopic children*Intervention = −0.20 ± 0.69Control = −0.37 ± 0.67*P* = 0.125	Not available	Intervention = 8.41%Control = 17.65%*P* < 0.001	Not available

RCT, randomized controlled trial; RLRL, repeated low-level red-light therapy; RWS, real-world survey; SVS, single-vision spectacle.

Only 2 out of 13 studies investigated the rebound effect of red-light therapy after cessation of treatment. Chen et al., in 2023, reported that AL and SER significantly increasing (*P* < 0.05) by 0.16 mm (95% confidence interval [CI] = +0.11 to +0.22 mm) and −0.20 D (95% CI = −0.26 to −0.14 D) over an observed period of 3 months after cessation of treatment, whereas also reporting that the increase in AL and myopic refraction was faster after therapy cessation; subfoveal CT also returned to baseline measurements in the intervention group after treatment cessation.^22^ Xiong et al., in 2022, reported an axial elongation of +0.42 mm (*P* < 0.001) and an SER progression of −0.91 D (*P* < 0.001) being observed over 12 months after the cessation of red light treatment. This rate of myopia progression compared to the control group was reported to be greater than the observed second year progression (AL = +0.28 mm, *P* = 0.005 and SER = −0.54 D, *P* = 0.12) but similar to the first-year progression in the control group (AL = +0.38 mm, *P* = 1.0 and SER = −0.71 D, *P* = 0.93), suggesting a modest rebound effect after cessation which did not completely obliterate the benefits conferred by the preceding red-light therapy. A decrease in red-light therapy efficacy in the second year of treatment compared to the first year was also reported: the red light treatment efficacy in reducing axial elongation dropped from 89.5% in the first year (repeated low-level red-light therapy for the first and second year [RLRL−RLRL] = +0.04 mm, control group [SVS−SVS] = +0.38 mm) to 57.1% in the second year (RLRL−RLRL = +0.12 mm and SVS−SVS = +0.28 mm) and similarly for slowing SER progression efficacy dropped from 84.5% (RLRL−RLRL = −0.11 D and SVS−SVS = −0.71 D) in the first year to 63.0% (RLRL–RLRL = −0.20 D and SVS–SVS = −0.54 D) in the second year^30^ (see Table 2).

Lin et al., in 2023, reported that red-light therapy yielded a significantly greater decrease in AL in children with a baseline AL of more than 24 mm. In fact, AL change in participants with an AL greater than 24 mm at baseline was −0.19 ± 0.13 mm, whereas the AL change in participants with a baseline AL less than 24 mm was −0.02 ± 0.07 mm (*P* = 0.007)^27^ after 60 days of red-light therapy*.* One study reported no significant difference in changes in AL and SER between genders^34^ and three studies reported no significant difference in changes in AL and SER^27^^,^^34^ and myopia incidence^23^ between different age groups.

Two studies reported potential dose-response efficacy in myopia control with therapies that emit red-light at 0.03 mW^24^ and 0.37 mW,^31^ with the study by Zhou et al., in 2024, finding that among 0.37 mW, 0.60 mW, and 1.20 mW powers of red-light therapy, all powers have a significant effect on myopia progression and control with no statistically significant difference in efficacy between the different powers^31^ (see Table 2).

### Safety

Eight out of 13 studies^22^^–^^25^^,^^29^^–^^31^^,^^34^ reported no adverse effects after continued red-light therapy. Three studies did not assess adverse effects related to red-light therapy.^28^^,^^32^^,^^33^ Two studies reported adverse effects which resolved spontaneously^26^^,^^27^ (Table 2). One study reported no adverse safety effects after therapy, but instead reported two participants withdrawing due to bright light intolerance.^25^

**Table 3.** Implementation Outcomes

Author, Year; Study Design; Sample Size	% of LTFU + Reasons	Compliance Rate	Implementation Factors: Drivers and Strategies	Implementation Factors: Barriers
He et al., 2023^23^; RCT; *N* = 278	10.8% (30/278); lost to follow up (COVID-19), withdrew	Median compliance rate of intervention group: 60.0% (IQR = 54.2%–64.8%)	Training of parents/guardians to supervise intervention, assignment of investigators to monitor intervention compliance, weekly reminders about the intervention	Participants did not bring the devices home from school during the COVID-19 lockdown period
Chen et al., 2023^22^; RCT; *N* = 102	15.7% (16/102); lost contact, withdrew, turned to other myopia treatments	Not available	WeChat group to monitor compliance, uploading photographs of the LRL treatment to the WeChat group daily	Not available
Dong et al., 2023^24^; RCT; *N* = 112	3.6% (4/112); refusal to follow up, did not have at least one post-randomization follow-up time point	87.5% compliance rate in RLRL group and 94.5% compliance rate in sham device control groups	Reminders were sent to parents/guardians about intervention compliance	Not available
Jiang et al., 2022^25^; RCT; *N* = 264	14.8% (39/264); self-reported discomfort, refused to follow up, turned to other myopia treatments	Median treatment compliance rate of 75% (IQR = 14.1–112.1%)	Reminders were sent to parents/guardians about intervention	Not available
Liu et al., 2024^26^; RCT; *N* = 94	9.6% (9/94) lost to follow up, segmentation errors, poor image quality	Not available	Training of parents/guardians to supervise intervention, assignment of investigators to monitor intervention compliance, weekly reminders about the intervention, device was integrated with an automated diary function and connected to the internet	Not available
Lin et al., 2023^27^; RCT; *N* = 164	21.9% (46/210); refusal to follow up, did not meet the inclusion criteria, lost to follow up (COVID-19), lost to other myopia control methods	Not available	Supervision of intervention by parents/guardians	Not available
Yang et al., 2022^28^; RCT; *N* = 25	No LTFU	Not available	Not available	Not available
Wang et al., 2023^29^; non-randomized controlled trial; *N* = 49	10.2% (5/49); turned to other myopia treatments, COVID-19	Median therapy adherence rate of 75% (IQR = 88.89–94.44%)	Not available	Not available
Xiong et al., 2022^30^; post-trial follow up RWS of Jiang et al., 2022; *N* = 138	17.4% (24/138); turned to other myopia treatments, did not adhere to treatment protocol	Not available	Not available	Not available
Zhou et al., 2024^31^; RCT; *N* = 200	11.5% (23/200); too busy/inconvenient for participants to follow up, lost contact, COVID-19, withdrawal, concern over side effects, worsening myopia, turned to other myopia treatments	Not available	Not available	Not available
Zhao et al., 2023^32^; non-randomized controlled trial; *N* = 67	No LTFU	Not available	Not available	Not available
Xiong et al., 2021^33^; RCT; *N* = 200	26% (52/200); explore other myopia control methods, no time for aftercare, not following doctors’ instructions, lost to follow up	Not available	Not available	Not available
Tian et al., 2022^34^; RCT; *N* = 224	20.1% (45/224); discontinued treatment, turned to other myopia treatments, lost contact	Not available	Not available	Not available
Mori et al., 2021^35^; RCT; *N* = 113	19.5% (22/113); using other myopia treatments, did not spend at least 1 h outside daily, excludable baseline characteristics, withdrawal due to participant issues and family issues	Not available	Not available	Participants did not have sufficient outdoor time exposure
Torii et al., 2022^36^; RCT; *N* = 43	18.6% (8/43); did not receive intervention, discontinued intervention from not satisfying study design, dropped out due to concern over myopia progression, participant was taking an excluded medicine, discontinued from fragile eyeglasses	Not available	Irradiation time was recorded by violet light frames and saved to a cloud server involving software installed on an iPod touch	Trial was initially suspended, and three participants withdrew due to the fragility of experimental and pseudo-placebo violet-light emitting eyeglass frames
Hua et al., 2015^37^; RCT; *N* = 317	No LTFU	Not available	Average illuminance of desks after the intervention surpassing the 300 lux requirement and the median uniformity of desk lighting increasing to 0.67	Intervention was not consistent regarding the illuminance and uniformity of blackboards - only one light fixture was added to just one half of the blackboard as the other half was used for multi-media presentationsAverage illuminance was achieved on only 88% of the recommended value for blackboards in the intervention group and significantly lower than the control arm
He et al., 2022^19^; RCT; *N* = 6295	19.5% (1228/6295); refusal to accept cycloplegia, absent, transferred schools	Not available	Approval and support from the Shanghai Education Bureau and Shanghai Health Bureau, issuance of an official statement inviting the schools and eye health departments to participate in and support the intervention program, supervision of intervention at various levels (e.g. school, district, and municipal), recording of information using a web-based application, assignment of investigators to monitor intervention compliance, assignment of a wearable wrist-watch light sensor to participants	Physical space availability, opportunity for structured activities, cultural attitudes on sun exposure and academic performance, weather (e.g. pollution) and short duration of breaks may have limited chances for children to be outdoor while on breakNumerous breaks for the additional 80 min intervention required multiple transitions from outdoor to classroom, which were made difficult by multi-story design of buildings
Wu et al., 2018^20^; RCT; *N* = 930	25.5% (237/930); excluded due to presence of other myopia treatments but still attended baseline examinations, did not attend final follow up assessment	At final assessment, compliance of participants was 86% in the intervention group and 88% in the control group	Assignment of a wearable wrist-watch light sensor to participants during school time and a diary log to track outdoor time outside of school time	Changing climate in different regions of Taiwan
Wu et al., 2013^21^; non-randomized controlled trial; *N* = 571	No LTFU	Not available	Not available	Not available

LTFU, loss to follow-up; RCT, randomized controlled trial; RWS, real-world study.

### Implementation

Four out of the 13 studies focusing on red-light therapy as an intervention reported compliance rates (i.e. a form of “fidelity of receipt” assessment) to red-light therapy,^23^^–^^25^*^,^*^29^ with compliance rates ranging from 60% to 94.5%*.* The study by He et al., in 2023, attributed the cause of their low median compliance rate to be from participants forgetting to bring the red-light devices home during the coronavirus disease 2019 (COVID-19) lockdown period.^23^ Strategies to monitor and increase intervention compliance involved linking the intervention device to the internet accompanied by an automated diary function which recorded treatment history,^25^^,^^26^ tracking the attendance of participants for treatment sessions, and sending reminders to study personnel who subsequently contacted the participants’ parents or legal guardians to facilitate improvements in treatment compliance,^23^^–^^25^^,^^29^^,^^30^ creation of a WeChat group tracked by a researcher where participants were required to upload photographs of their treatment sessions daily to ensure compliance^22^ and training of guardians or parents to supervise the intervention at home^23^ (see Table 3). No data were reported on the effect of these strategies on compliance rates.

The studies by Wang et al., in 2023, and Jiang et al., in 2022, reported a significant positive correlation between treatment compliance and treatment efficacy. The study by Wang et al., in 2023, reported that as therapy compliance increased from less than 50% to more than 75%, efficacy in delaying AL growth significantly increased from 37.5% to 188%, and efficacy in increasing subfoveal CT significantly increased from 91.9% to 117%, with *P* values < 0.001.^29^ Jiang et al., in 2022, reported that with improvements in treatment compliance from less than 50% to more than 75%, efficacy significantly increased from 44.6% to 76.8% in reducing axial elongation, and from 41.7% to 87.7% in controlling SER progression, with *P* values < 0.001.^25^

We further analyzed loss to follow up as an indicator of intervention implementability, within the trial context. Across all 13 studies which focused on red-light therapy as an intervention, the total loss to follow up ranged from 0% to 26%, with a median loss to follow up of 14.8%.^22^^–^^34^ Participants were lost to follow up due to the COVID-19 lockdown,^23^^,^^27^^,^^29^^,^^31^ turning to other myopia treatments,^22^^,^^25^^,^^27^^,^^29^^–^^31^^,^^33^^,^^34^ deviation from treatment protocol,^30^*^,^*^33^ and no time for aftercare,^33^ self-reported discomfort,^25^ segmentation errors and poor image quality upon outcome measurement,^26^ not meeting the study inclusion criteria,^27^ concern over side effects,^31^ worsening myopia leading to withdrawal in the control group,^31^ deviation from treatment protocol,^30^*^,^*^33^ and no time for aftercare.^33^ These reasons for loss to follow up were reported in both the intervention and control groups. The loss to follow up based on turning to other myopia treatments in studies ranged from 2.9% to 7.8%, whereas loss to follow up based on concern over side effects was only reported in one study as being 1.7%.

No studies investigating red-light therapy reported any evidence nor described any reliable measurements regarding any other standard implementation outcomes – including perceived acceptability, intended adoption of interventions by stakeholder groups, costs of implementation, sustainability, or maintenance of the red-light therapy intervention over time.

## Violet/Ultraviolet Light Therapy

### Effectiveness

No significant differences in AL and SER were observed between the intervention and control groups with violet/UV light therapy (360–400 nm) before specific subgroup analysis in both studies by Mori et al., in 2021,^35^ and Torii et al., in 2022.^36^ After specific subgroup analysis, the study by Mori et al., in 2021, found that violet-light transmitting eyeglasses have a significant effect on axial elongation in children with no previous history of eyeglasses use and who have near-work time less than 180 minutes daily.^35^ In contrast, the study by Torii et al., in 2022, found that violet/UV light emitting eyeglasses (360–400 nm) significantly reduce changes in cycloplegic SER and AL elongation in children aged 8 to 10 years.^36^

### Safety

Adverse effects, such as abnormalities in retinal or choroidal structure and dermopathy, were evaluated in both studies. However, none of the adverse effects reported were specifically associated with violet light therapy.

### Implementation

The study by Mori et al., in 2021, reported a 19.5% loss to follow up rate due to familial issues and protocol deviation.^35^ The study by Torii et al., in 2022, reported a 18.6% loss to the follow up rate due to personal and family issues with the eyeglasses – 6.9% (3/43) of the total participants were lost to follow up due to fragile eyeglasses. The violet-light emitting eyeglasses trial was initially suspended due to the fragility of the eyeglass frame and was resumed after the frame was upgraded with a stronger material^36^ (see Table 3). Methods used to monitor and increase compliance, although compliance rates were not reported, included tracking of violet light irradiation through recording on software connected to a cloud server.^36^ Neither study report any evidence related to any other implementation outcome.

## Increased Classroom Illumination

### Effectiveness, Safety, and Implementation

The study by Hua et al., in 2015, reported a reduction in the prevalence of new onset myopia in the intervention group (4%) compared to the control group (10%; *P* = 0.03). Among patients without myopia, in the intervention group compared with controls, changes in both mean SER (−0.25 ± 0.40 D vs. −0.47 ± 0.40 D, *P* = 0.001) and AL (+0.13 ± 0.17 mm vs. +0.18 ± 0.12 mm, *P* = 0.02) were significantly reduced. However, among myopic children between the intervention and control groups, the mean change in SER between the two groups was not significantly different (−0.25 ± 0.47 vs. −0.31 ± 0.46 D, *P* = 0.39), although the mean axial elongation was significantly shorter in the intervention group compared with the controls (+0.20 ± 0.11 mm vs. +0.27 ± 0.10 mm, *P* = 0.0001; see Table 2). No safety outcomes were assessed in this study (see Table 2). Increased classroom lighting was reported with the median average illuminance of desks increased up to 558 lux (interquartile range [IQR] = 506–603 lux) after the intervention, surpassing the 300 lux requirement, with the median uniformity of desk lighting increasing to 0.67 (IQR = 0.64–0.71) approaching the recommended value of 0.7. However, due to practical constraints, the median average illuminance of blackboards only increased to 440 lux, 88% of the recommended level of 500 lux, with uniformity declining to 0.65 (IQR = 0.59–0.71) from a pre-intervention value of 0.72 (IQR = 0.62–0.75). A loss to the follow up rate of 13.7% due to participants being excluded from other simultaneous myopia treatment or eye disease, lack of eligibility for cycloplegia, and being unwilling to travel to the hospital for an eye examination^37^ (see Table 3). No data about any other outcome were reported.

## Increased Exposure to Light Outdoors

### Effectiveness

Three studies reported a significant reduction in myopic SER, AL elongation, and reduced myopia incidence between the intervention and control groups.^19^^–^^21^ However, within the selected studies in this review, evidence is uncertain whether this effect is significant in both patients without myopia and patients with myopia^20^ or patients without myopia only^19^^,^^21^ (see Table 2). The study by Wu et al., in 2018, also reported in their subgroup statistical analysis that participants who had 200 minutes or more of weekly outdoor time during school and were not myopic at baseline had significantly less myopic shift when exposed to moderate light intensity in environments of 1000 lux (+0.18 D, 95% CI = 0.04–0.32, *P* = 0.01) or more, 3000 lux or more (+0.22 D, 95% CI = 0.06–0.37, *P* = 0.006), or 5000 lux or more (+0.24 D, 95% CI = 0.14–0.33, *P* < 0.001). However, when assessing participants who had 125 to 199 minutes of outdoor time during school, only those without myopia at baseline who were exposed to a 10,000 lux or more had significantly less myopic shift (+0.16 D, 95% CI = 0.08–0.24, *P* < 0.001). Thus, the study by Wu et al., in 2018, suggested that non-myopic school children who are exposed to less than 200 minutes of outdoor time per week may need exposure to environments with illuminations greater than 10,000 lux to achieve protective effects against myopia, whereas in those who have at least 200 minutes of weekly outdoor time, moderate light intensity environments greater than 1000 lux may be sufficient to protect against myopia.^20^ The study by Wu et al., in 2013, noted that combined treatment of outdoor activity and atropine did not further control myopia progression.^21^

### Safety

None of these studies assessed or reported any safety outcomes related to eye health associated with outdoor time (see Table 2).

### Implementation

The study by Wu et al., in 2018, reported a compliance rate of enrolled participants as high as 100%, however, when excluding participants who did not attend the final follow-up assessment, they reported 86% and 88% compliance rates in the control and intervention groups, respectively^20^ (see Table 3). Noncompliance occurred in the intervention groups, with the control group also potentially receiving the intervention being reported.^19^^,^^20^ The study by He et al., in 2022, reported no compliance rates, however, it was mentioned that implementation of outdoor time was achieved for 84.6% of the intervention group with an extra 40 minutes of outdoor time per school day and 88.0% of the intervention group with an additional 80 minutes of outdoors time per school day^19^ (see Table 3). The study by Wu et al., in 2013, reported no implementation outcomes. The studies by He et al., in 2022, and Wu et al., in 2018, reported moderately high loss to follow up rates (see Table 3) with the reasons for the loss to follow up generally being due to the design of the study and external factors (such as transferring schools) rather than the implementation of increased outdoor time. The study by Wu et al., in 2013, reported no loss to follow up. Methods to monitor and improve compliance include approval and support from government agencies, supervision of intervention at various levels (e.g. school, district, and municipal), recording of information using a web-based application, assignment of investigators to monitor intervention compliance, and assignment of a wearable wrist-watch light sensor^19^ and self-recorded diary log to participants to track light exposure^20^ (see Table 3).

## Discussion

To our knowledge, this is the first systematic review to not only synthesize effectiveness and safety, but also implementation evidence for light therapies for myopia prevention and control in children. Our review found high quality evidence across multiple randomized controlled trials and non-randomized interventional studies showing red-light therapy having significant effectiveness on the alleviation of AL elongation and myopic SER increases, in addition to increased CT in the intervention groups compared with the control groups, after a therapy period ranging from 4 weeks to 24 months – a finding consistent with recent meta-analyses reporting that red-light therapy may be effective in alleviating myopia progression through reducing axial elongation and myopic refraction changes,^39^^,^^40^ and through increasing CT.^38^^,^^79^ Nonetheless, despite the extensive evidence supporting the effectiveness of red-light therapy, only one study^30^ investigated its effects over a period longer than 12 months. Other studies also report red-light therapy as having a greater effectiveness compared with other treatments^33^^,^^73^ with a rebound effect potentially related to its high efficacy.^30^ This rebound effect upon cessation may lower the clinical usefulness of red-light therapy through reduced sustainability of its effects, however, the limited pool of evidence makes it difficult to draw consistent, definitive conclusions about these effects. Furthermore, with evidence being uncertain regarding the effectiveness of red-light therapy between children of different age groups and with different myopia severities, it can be concluded that more studies should be conducted to investigate the effects of red-light therapy over a period of at least 2 years, its efficacy between different age groups and myopia severities, its efficacy compared with other myopia treatments, and its associated rebound effect.

Conflicting evidence regarding the safety of red-light therapy was also found, with two studies reporting minor adverse effects which resolved spontaneously. However, the long-term adverse effects of red-light therapy beyond a continued 2-year treatment period remains unknown, and safety concerns related to the intensity and duration of the interventions have recently been raised by a study by Ostrin et al., in 2024.^80^ It is worth noting, however, that Ostrin's conclusions may not apply to all red-light devices.^81^ The uncertain long-term safety evidence for red-light therapy makes it difficult to definitively conclude whether the benefits and effectiveness of this therapy outweigh the risks associated with its administration. Evidence was, however, found supporting significant effectiveness of myopia control at lower powers than the currently adopted red-light therapy regimen. Thus, whereas further investigations should be conducted to confirm the long-term side effects of continuous red-light therapy and ensure the safety of all commercially available red-light therapy devices, a treatment regimen with a more conservative power can be considered to both control myopia progression and alleviate any potential long-term side effects.

Our review found that implementability of light therapies for myopia is overall under-researched. Fidelity of therapy receipt, in the form of compliance, was the most reported implementation measure. This was found highly variable across studies – for example, ranging from very low (14.1%) to very high (112.1%) for red-light therapies, with little data explaining the reasons behind this range. Possible strategies to support or enhance implementation with users (i.e. children and their families/guardians) were reported but without direct evidence on how effective these were in maintaining or increasing compliance with red-light therapy delivery. The small and solely descriptive amount of implementation data makes it difficult to draw definitive conclusions on the fidelity of red-light therapy. Furthermore, considering evidence for a positive adherence-response relationship was reported, we conclude that strategies to improve compliance and possible barriers to compliance of red-light therapy should be investigated systematically. To support the widespread adoption of red-light therapy as a treatment for myopia prevention and control in children, future studies researching red-light therapy should plan in their protocols to record compliance rates, assess the effect between different methods to increase compliance, and also confirm the positive adherence-response relationship identified by this review.

Some indirect implementation evidence for red-light therapy can be deduced from reported loss to follow-up rates. Even within the study context (i.e. not the usual setting for these children or their parents/guardians), such evidence informs the adoption and/or reach potential of red-light therapy. Participants that withdrew specifically from self-reported discomfort caused by the red-light therapy or concern over associated side effects explained a small proportion of the total loss to follow up in the respective studies. Studies that reported participants turning to other myopia treatments reported no data about whether this was due to the red-light therapy intervention or for unrelated reasons – more detailed reporting of reasons for loss to follow up in future studies could help identify factors affecting the perceived acceptability and intended adoption of red-light therapy. It should also be noted that the reported loss to follow-up rates also included methodological factors specific to the individual study design rather than the red-light intervention which can result in overestimation of its effect on implementation.

There is existing evidence to suggest that red-light therapy is less cost effective than other existing myopia treatments which may be a barrier to its implementation.^82^ However, this study did not account for the potential of rebound when calculating the cost effectiveness of treatments, with red-light potentially being even more costly with its potentially greater relative rebound effect.

Compared to red-light therapy, this review found very limited evidence supporting a significant effect of violet/UV light therapy on myopia prevention and control. Specific subgroup analysis undertaken by the violet light studies should be taken with caution due to the small sample size used in both studies’ analyses and should be confirmed with studies with a larger sample size. A previously published case report has also described axial shortening and choroid thickening in a 4-year-old child after 24 months of wearing violet light transmitting eyeglasses and having 2 hours of outdoor activities daily, with no adverse effects being observed,^83^ still indicating potential for violet light to be a significantly effective treatment for myopia control.

None of the included studies investigating violet light reported any adverse effects from violet-light exposure, nonetheless, researchers and clinicians should still note the risks of excessive ultraviolet light exposure namely immunosuppression,^84^ premature skin aging,^85^ and macular degeneration.^86^^,^^87^ Additionally, some studies suggested a link between violet light, and potential retinal damage,^88^ such as chorioretinal atrophy and visual loss.^89^

Both violet light studies failed to report compliance rates and provided limited reasons behind loss to follow-up rates, making it difficult to draw any conclusions about the fidelity of delivery of violet-light interventions. Nonetheless, as violet-light transmitting eyeglasses need a minimum outdoor time to work effectively, this could represent a potential barrier to implementation in environments where children have limited outdoor time. At the same time, there is potential to complement outdoor time treatment regimens with the use of violet-light emitting eyeglasses indoors to improve myopia treatment efficacy, however the fragility of the currently proposed design for violet-light emitting eyeglasses presents a moderate concern and barrier to its implementation. The cost effectiveness of violet-light transmitting eyeglasses has yet to be and should be investigated to further support implementation discussions. Evidence regarding use of violet light to control and prevent myopia is still in its infancy thus more studies should be conducted to confirm the efficacy, safety, and rebound of violet light, as well as to enable discussions about perceived acceptability and sustainability of violet-light therapy.

Regarding the effect of improved indoor classroom lighting on myopia prevention and control, only one cluster randomized controlled trial reported significant effectiveness – similar to a previous observational study^90^ – however, this effectiveness was reported to be varied between patients without myopia and patients with myopia. The study by Hua et al., in 2015, noted decreased illuminance at blackboards due to practical constraints, highlighting an important barrier to feasibility. No loss to follow up related to the implementation of indoor classroom lighting was also found, which may be due to the institutional, group-based, and noninvasive nature of the intervention generally raising light levels for all students and not specifically increasing light exposure to an individual, reducing the risk of both adverse effects and implementation challenges. Data regarding rates of compliance to the intervention were not reported, however, the regimented nature of school attendance as opposed to implementation of interventions at home may have allowed for higher compliance rates to the improved indoor classroom lighting environment. Further research is warranted to replicate and directly test these observations for indoor classroom lighting.

Corroborating previously published literature, our systematic review found that increased outdoor time had significant effectiveness in myopia prevention and control. However, conflicting evidence emerged regarding the significant effects of increased outdoor time on changes in SER and the effect of outdoor time on patients with myopia and patients without myopia. Nonetheless, current meta-analyses show that outdoor time significantly reduces SER change in intervention groups compared with controls^91^^,^^92^ and that outdoor time is more effective in patients without myopia, but still generally beneficial for myopia control in patients with myopia.^4^ No outdoor time studies included in this review recorded adverse effects related to increased outdoor time, however, the risks of outdoor exposure are well documented and should be recognized by researchers and clinicians alike. These risks include sunburns^93^ and increased risk of skin cancers.^94^ In children specifically, exposure to dangerous creatures, poisonous plants, allergens, and irritants, such as bug bites, are some risk factors associated with increased outdoor time.^95^^,^^96^ These naturally existing risks should be considered in conjunction with the benefit of increased outdoors time for myopia control.

Evidence on implementation of outdoor time was sparse. The study by He et al., in 2022, that did report on those identified some challenges, despite the regimented nature of schools which might be expected to aid implementation. Challenges could have been due to the implementation of increased outdoor time being hindered by physical space availability, opportunity for structured activities, weather, and logistical issues involving short break times and transitions between outdoors and classrooms, differing daily regional sunlight hours and cultural attitudes on sun exposure and academic performance.^19^ A high compliance rate was found for outdoor time in the sole relevant study included in this systematic review indicating potential for successful implementation, whereas also pointing toward a need for more studies which focus on outdoor time to report compliance rates.

### Limitations

Our review has limitations. First, implementation evidence was sparse and limited in nature when available, precluding firm conclusions about scalability and sustainability of the review therapies. Second, only studies in English were included in this review, which may have omitted potentially relevant research.

### Novel Ideas and Extensions (Limitations of Research Space)

Behavioral light therapy strategies to prevent and control myopia in children, such as increased outdoor time, have been well documented and researched, however, research into the effectiveness and implementation of technical light therapies (red-light and violet light emitting devices, and improved indoor classroom lighting) is still in its infancy. Current scientific literature shows that red-light therapy has been proven to have significant effectiveness at myopia prevention and control, but few studies have explored strategies to improve implementation of red-light therapies and monitored the degree of implementation success, unlike outdoor time. Similarly, research surrounding the effectiveness of red-light therapy after periods of treatment longer than 2 years is still underdeveloped, along with evidence showing effectiveness of red-light therapy in different subgroups of children. Violet light therapies and improved indoor classroom lighting have the potential to be used for myopia prevention and control, but more research should first be conducted to determine their effectiveness and potential for successful implementation in larger populations. Other light therapies which have potential to control myopia but have yet to be explored in an interventional study that includes the use of a novel bright classroom to generate a greater light intensity environment^68^ and the effect of exercise supplementing the myopia control effect of outdoor time.^75^ As more research is to be conducted on the impact of light delivery devices and ambient light on myopia, these should consider the standardization in reporting of protocol and light intervention to allow for better replication and meta-analyses.^97^

Evidence exists to show that combining increased outdoor time with atropine treatment did not have a synergistic effect,^21^ however, existing research has yet to show the combined effect of different light therapy regimens. A similar approach can also be taken toward investigating the combining of light therapies with other existing non-light strategies to control myopia – given the reduced efficacy of red-light therapy in the second year of therapy and potential rebound effects of existing myopia treatments, the possibility exists for combinations of light therapies and traditional myopia control strategies in children to achieve maximum efficacy of myopia control.

It is important to note that all light therapies discussed in this review impose different levels of treatment burden on both children and their families. This burden, which may involve significant time commitments or logistical planning, could pose a barrier to widespread implementation. The extent to which this burden affects the overall effectiveness and benefits of these therapies remains unclear and warrants further investigation. Future studies should focus on exploring feasible implementation approaches for light therapies aimed at controlling and preventing myopia progression.

Most of the studies included in this review involved Chinese participants, with the exception of two studies that focused on Japanese children, underlining a need to carry out similar studies with other nationalities. Large-scale, multiethnic studies with planned subgroup analysis should be conducted in order to fill in these research gaps.

### Implications for Policy and Practice

Although evidence exists showing the significant effectiveness red-light therapy has on preventing and controlling myopia progression, with a very limited number of studies showing the significant effectiveness over a treatment period greater than 2 years, red-light therapy may not yet be suitable to be recommended for widespread use, especially because myopia can continue to develop from ages 5 to 16 years.^98^^–^^100^ Similarly, the potential modest rebound effect upon cessation of red-light therapy shows a potential lack of sustainability and potential futility if children or their parents/guardians are unable or unwilling to pursue red-light treatment, hence opt to stop it prematurely. To minimize this rebound effect, a gradual reduction in treatment sessions over time are likely beneficial, as has been suggested for atropine.^30^ Switching children into alternative therapy strategies may also alleviate the rebound effect. Existing approaches currently reported by studies included in this review can be used as a reference for strategies to aid implementation of red-light therapy. However, with safety concerns, moderately high but wide-ranging compliance rates, and other more cost-effective alternatives, red-light therapy has some evidence to suggest potential challenges in scaled implementation. In contrast, strategies to control myopia by increasing outdoor time have already been implemented in primary schools in Singapore,^42^ Taiwan,^101^ and China^102^ with successful effects.

## Supplementary Material

Supplement 1
