# Supplementary material for: Light Therapy for Myopia Prevention and Control: A Systematic Review on Effectiveness, Safety, and Implementation
Source: Transl Vis Sci Technol. 2024 Aug 21;13(8):31. doi: 10.1167/tvst.13.8.31 (PMC11343011; doi:10.1167/tvst.13.8.31)
Supplement: Supplement 1 [file tvst-13-8-31_s001.docx]

**Supplementary material**


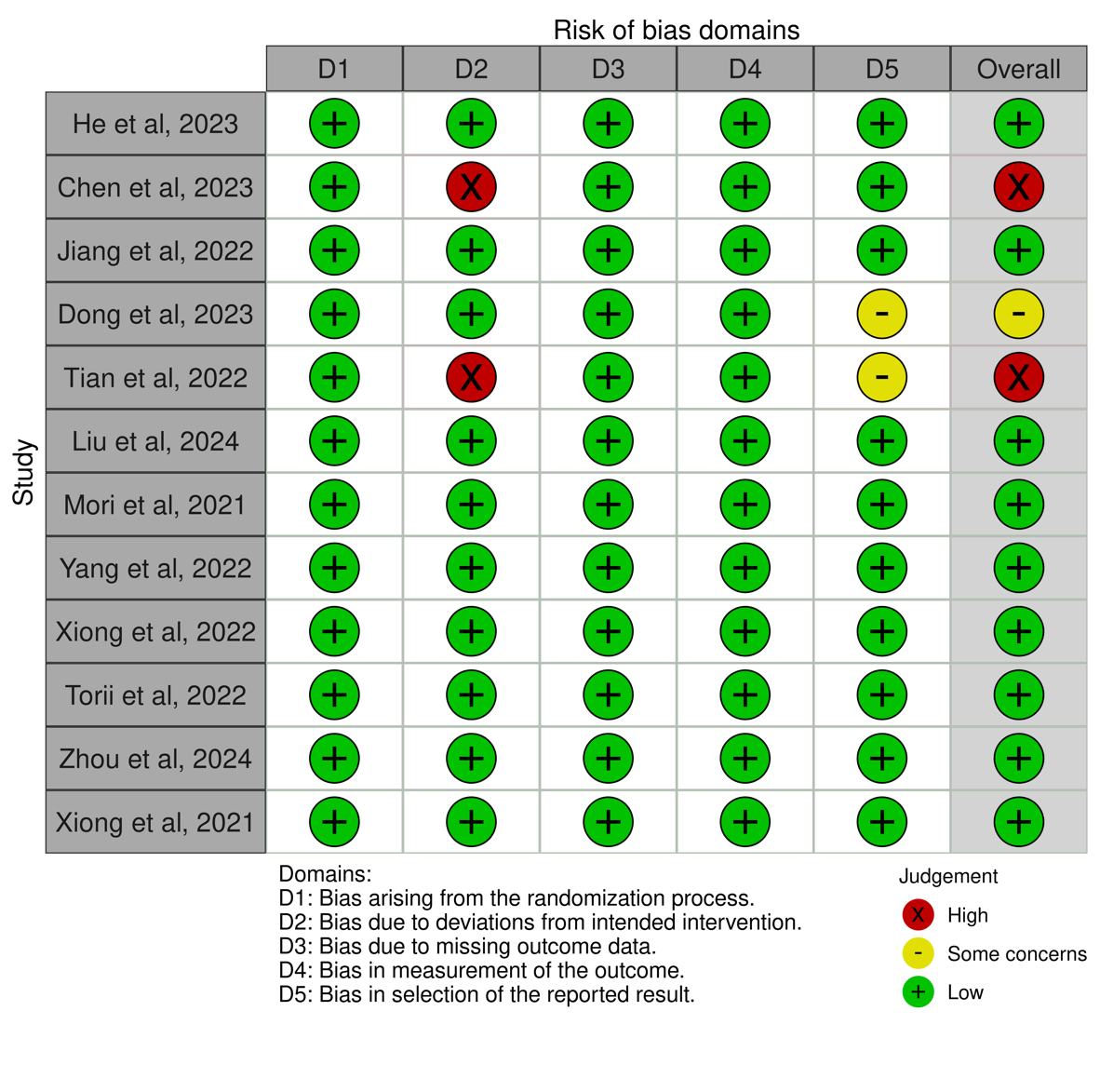


**Figure S1.** Risk of bias in selected randomised controlled trials (RCTs).


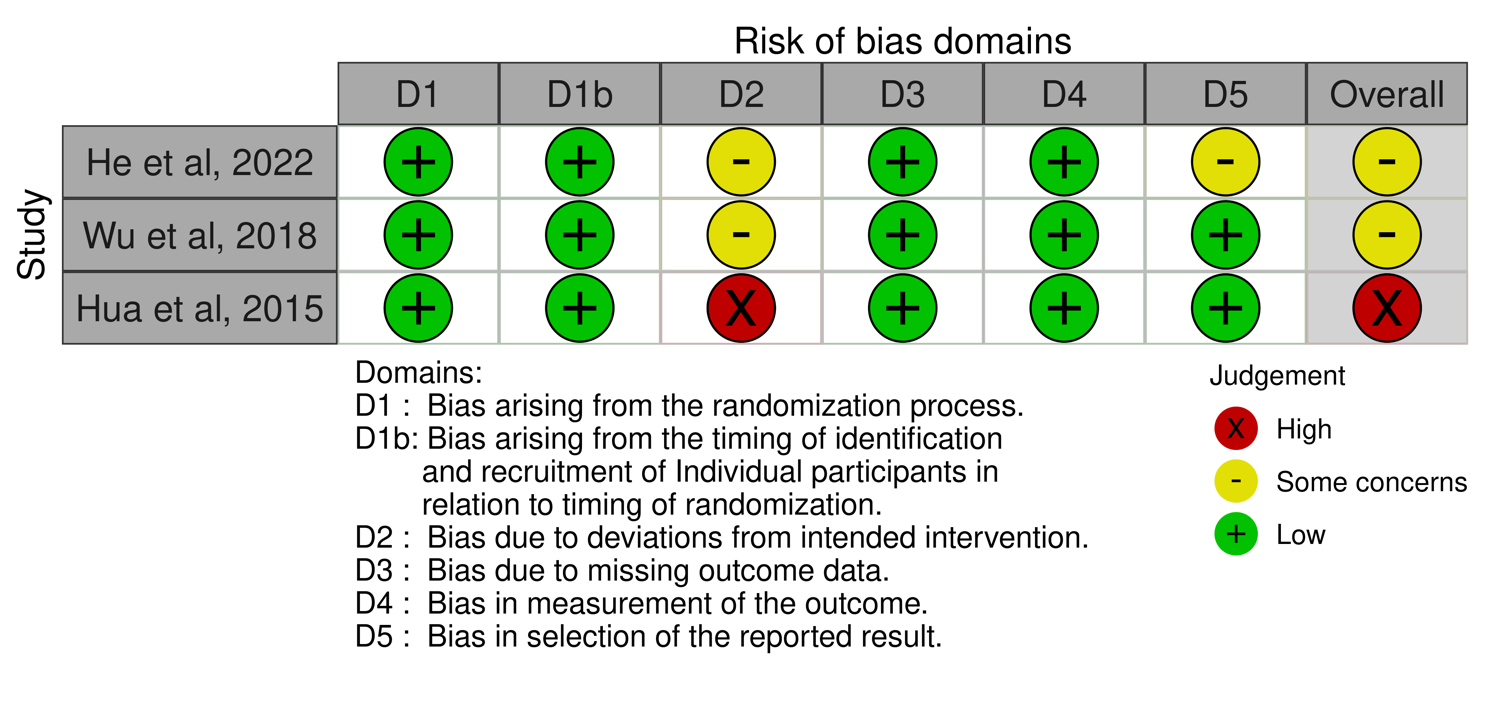


**Figure S2.** Risk of bias in selected cluster RCTs.


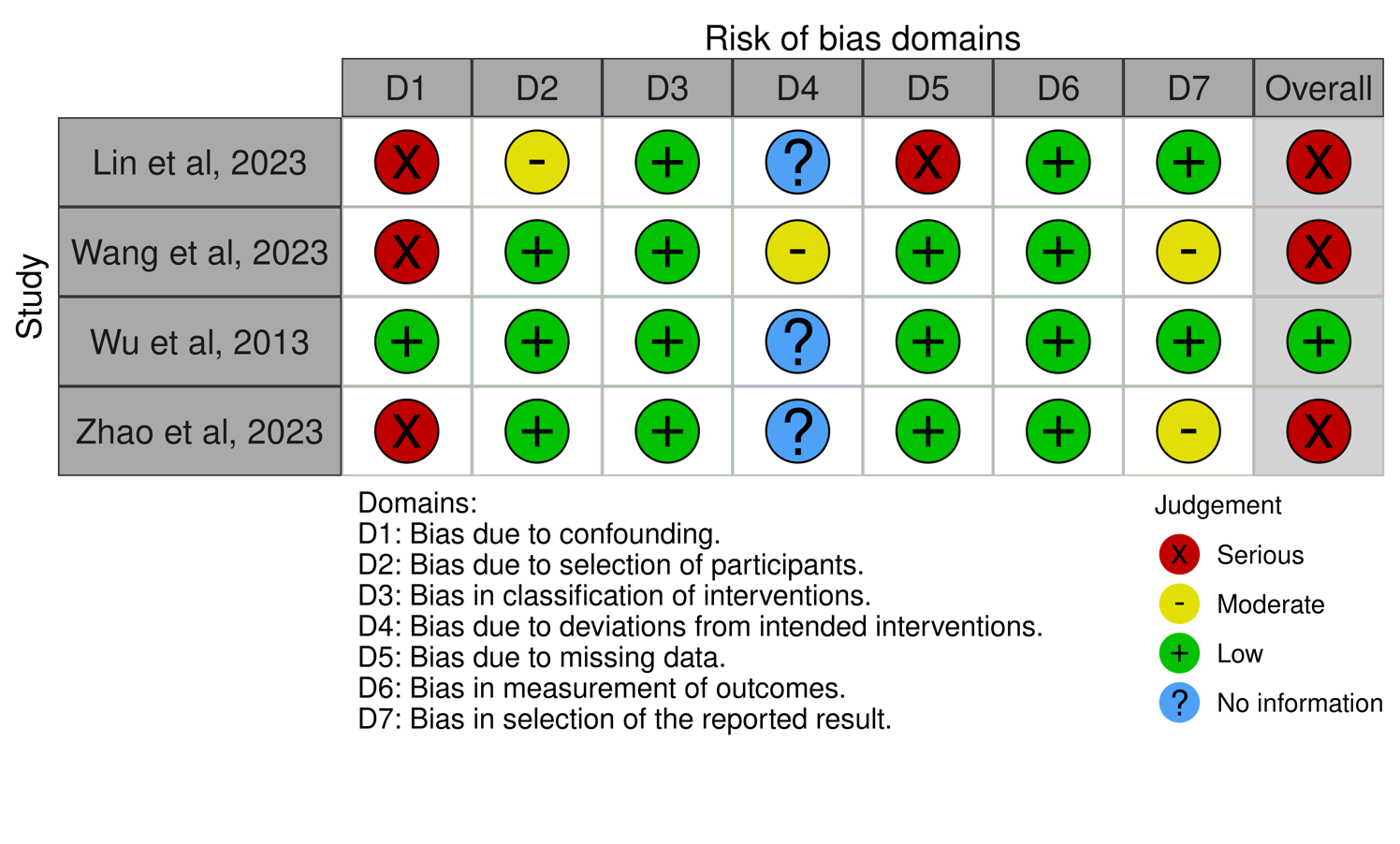


**Figure S3.** Risk of bias in selected non-randomised interventional studies.

**Table S1.** Tables of search terms for each database

**Pubmed:**

|  | ncbi filters used - English & Humans |  |
| --- | --- | --- |
| # | search term | no. of results (filtered) |
| #1 | Myopi* OR ((near[Title/Abstract] OR short[Title/Abstract] OR close[Title/Abstract]) AND (sight*[Title/Abstract] OR vision[Title/Abstract])) OR "axial length"[Title/Abstract] | 35 564 |
| #2 | light*[Title/Abstract] OR infrared*[Title/Abstract] OR ultraviolet*[Title/Abstract] OR laser*[Title/Abstract] OR illumina*[Title/Abstract] OR luminance*[Title/Abstract] OR LED[Title/Abstract] OR lux[Title/Abstract] OR (light*[Title/Abstract] AND (exposure[Title/Abstract] OR level[Title/Abstract] OR outdoor*[Title/Abstract] OR indoor*[Title/Abstract] OR spectrum*[Title/Abstract] OR environment[Title/Abstract] OR therapy[Title/Abstract])) | 774 247 |
| #3 | Child*[Title/Abstract] OR adolescent[Title/Abstract] OR teen*[Title/Abstract] OR student*[Title/Abstract] OR school*[Title/Abstract] OR juvenile[Title/Abstract] OR youth[Title/Abstract] OR young*[Title/Abstract] OR kid[Title/Abstract] | 2 068 864 |
| #4 | #1 and #2 and #3 | 997 |
| #5 | ("Myopia” [Mesh]) | 16 988 |
| #6 | "Phototherapy/methods"[Mesh] OR "Sunlight"[Mesh] OR "Low-Level Light Therapy"[Mesh] OR "Ultraviolet Rays"[Mesh] OR "Infrared Rays"[Mesh] | 59 326 |
| #7 | (("Child"[Mesh]) OR "Child Health"[Mesh]) OR "Adolescent"[Mesh] | 2 791 120 |
| #8 | #5 and (#6 OR #7) | 5126 |
| #9 | #5 and #6 and #7 | 42 |
| #10 | #4 or #9 | 1,023 |

**Embase:**

| # | search term | no. of results |
| --- | --- | --- |
| #1 | (myopi* OR ((near OR short OR close) AND (sight* OR vision)) OR 'axial length'):ti,ab AND [English]/lim | 51,670 |
| #2 | (light* OR infrared* OR ultraviolet* OR laser* OR illumina* OR luminance* OR led OR lux OR (light* AND (exposure OR level OR outdoor* OR indoor* OR spectrum* OR environment OR therapy))):ti,ab AND [english]/lim | 21,187,740 |
| #3 | (child* OR adolescent OR teen* OR student* OR school* OR juvenile OR youth OR young* OR kid*):ti,ab AND [English]/lim | 4,121,641 |
| #4 | #1 AND #2 AND #3 | 7,507 |
| #5 | #1 AND #2 AND #3 AND ([animal cell]/lim OR [animal experiment]/lim OR [animal model]/lim OR [animal tissue]/lim) | 897 |
| #6 | #1 AND #2 AND #3 NOT ([animal cell]/lim OR [animal experiment]/lim OR [animal model]/lim OR [animal tissue]/lim) | 6,610 |
| #7 | #1 AND #2 AND #3 AND ([child]/lim OR [preschool]/lim OR [school]/lim OR [adolescent]/lim) AND [humans]/lim AND [english]/lim | 756 |
| #8 | #1 AND #2 AND ([child]/lim OR [preschool]/lim OR [school]/lim OR [adolescent]/lim) AND [humans]/lim AND [english]/lim | 1205 |
| #9 | Emtree "Myopia" | 41,514 |
| #10 | Emtree "Phototherapy" | 37,266 |
| #11 | Emtree "illumination" | 62,527 |
| #12 | Emtree "Laser therapy" | 39,406 |
| #13 | Emtree "Ultraviolet phototherapy" | 1667 |
| #14 | Emtree "Ultraviolet radiation" | 123,408 |
| #15 | Emtree "Infrared radition" | 49,073 |
| #16 | #8 OR #9 AND (#10 OR #11 OR #12 OR #12 OR #13 OR #14 OR #15) | **2235** |

**CINAHL:**

| # | search term | no. of results |
| --- | --- | --- |
| #1 | TI = (Myopi* OR ((near OR short OR close) AND (sight* OR vision)) OR “axial length”) | 2594 |
| #2 | AB = (Myopi* OR ((near OR short OR close) AND (sight* OR vision)) OR “axial length”) | 5540 |
| #3 | TI = (light* OR photo* OR infrared* OR ultraviolet* OR laser* OR illumina* OR luminance* OR LED OR lux OR (light* AND (fluorescent OR wavelength OR exposure OR level OR outdoor* OR indoor* OR spectr* OR environment OR therapy))) | 58,203 |
| #4 | AB = (light* OR photo* OR infrared* OR ultraviolet* OR laser* OR illumina* OR luminance* OR LED OR lux OR (light* AND (fluorescent OR wavelength OR exposure OR level OR outdoor* OR indoor* OR spectr* OR environment OR therapy))) | 228,373 |
| #5 | CINAHL Subject Heading: MH "Myopia" | 3299 |
| #6 | CINAHL Subject Heading: MH "Phototherapy" | 3962 |
| #7 | CINAHL Subject Heading: MH "Lighting" | 2355 |
| #8 | CINAHL Subject Heading: MH "Light" | 4325 |
| #9 | CINAHL Subject Heading: MH "Sunlight" | 3633 |
| #10 | CINAHL Subject Heading: MH "Radiation, Non-Ionizing" | 972 |
| #11 | CINAHL Subject Heading: MH "Laser Therapy" | 10,756 |
| #12 | #5 AND (#6 OR #7 OR #8 OR #9 OR #10 OR #11) | 264 |
| #13 | (#1 AND #3) OR (#2 AND #4) | 1325 |
| #14 | #12 OR #13 | **1493** |

**Scopus:**

| # | search term | no. of results |
| --- | --- | --- |
| #1 | TITLE-ABS-KEY ("Myopi*" OR (("near" OR "short" OR "close") AND ("sight*" OR "vision")) OR "axial length") AND (LIMIT-TO (LANGUAGE, "English")) | 157,705 |
| #2 | TITLE-ABS ( "light*" OR "infrared*" OR "ultraviolet*" OR "laser*" OR "illumina*" OR "luminance*" OR "LED" OR "lux" OR "light*" AND "exposure" OR "level" OR "outdoor*" OR "indoor*" OR "spectrum*" OR "environment" OR "therapy" ) AND ( LIMIT-TO ( LANGUAGE , "English" ) ) | 1,755,216 |
| #3 | TITLE-ABS ( "Child*" OR "adolescent" OR "teen*" OR "student*" OR "school*" OR "juvenile" OR "youth" OR "young*" OR "kid*") AND ( LIMIT-TO ( LANGUAGE , "English" ) ) | 30,094,638 |
| #4 | #1 AND #2 AND #3 | 871 |
| #5 | #1 AND #2 AND #3 AND ( LIMIT-TO ( EXACTKEYWORD,"Human" ) ) | **536** |

**Web of Science:**

| # | search term | no. of results |
| --- | --- | --- |
| #1 | TS = (Myopi* OR ((“near” OR short OR close) NEAR/3 (sight* OR vision)) OR “axial length”) | 46,489 |
| #2 | TS = (light* OR photo* OR infrared* OR ultraviolet* OR laser* OR illumina* OR luminance* OR LED OR lux OR (light* AND (fluorescent OR wavelength OR exposure OR level OR outdoor* OR indoor* OR spectr* OR environment OR therapy))) | 10,383,910 |
| #3 | #1 AND #2 | 15,036 |
| #4 | TS = (Child* OR adolescent OR teen* OR student* OR school* OR juvenile OR youth OR young* OR kid*) | 5,801,902 |
| #5 | #1 AND #2 AND #4 | **2502** |
